# The referral centers for the diagnosis and treatment of hypertension in adolescents

**DOI:** 10.1186/1824-7288-41-S2-A14

**Published:** 2015-09-30

**Authors:** Giovanni Cerasola, Giuseppe Mulè, Santina Cottone

**Affiliations:** 1 European Society of Hypertension Excellence Centre of Palermo. Dipartimento Biomedico di Medicina Interna e Specialistica. Università di Palermo, Italy

## 

Primary hypertension in adolescence was felt to be quite rare. However, the worldwide childhood obesity epidemic has had a profound impact on the frequency of high blood pressure (BP) with the result that primary hypertension should now be viewed as one of the most common health conditions in the young (estimated prevalence 1–5%).

Therefore, current guidelines recommend that all children and adolescents seen in a medical setting should have their BP measured. The availability of BP tables with normal BP percentiles for age, sex and height has improved BP values classification.

Studies conducted at referral clinics for evaluation of hypertension have indicated that as many as 30 to 40% of adolescents may actually have in a clinical setting white-coat hypertension. This may lead to a misdiagnosis of “true” hypertension in a considerable number of cases. The usefulness of out-of-office BP evaluation using ambulatory or home monitoring is well established. These measurements allow the detection of the white-coat and masked hypertension, the opposite of white-coat hypertension, and are more closely associated with organ damage and cardiovascular risk than office measurements. A thorough familial and personal history is of primary importance as well as the physical examination that should be focused on the search for signs suggestive for an underlying cause and/or for the severity of hypertension.

Following investigations must be tailored to the child's age, anamnesis and clinical examination and to the severity of BP elevation, in order to investigate not only the possible cause of hypertension, but also associated diseases and target organs damage. Therapeutic approach should firstly include non-pharmacological measures, and the use of medications when indicated.

A key role in the management of the adolescents with hypertension may be attributed to the hypertension referral centers (table 1).

**Table 1:** What is required at the referral centers in the management of hypertension in adolescence

• To guarantee a multidisciplinary approach to the hypertension problem in adolescents. • To provide pediatric, cardiologic, nephrologic, endocrinologic, dietary and in some cases psychological expertise. • To obtain ample experience in the evaluation of organ damage, interpretation of 24 hour ambulatory blood pressure monitoring and self measurement of blood pressure at home. • To have access to laboratory techniques and instruments necessary for the diagnosis of different forms of secondary hypertension. • To build communication channels between pediatricians and family doctors with the aim of outlining the therapy and monitoring the adolescent with hypertension.

